# GraphADT: empowering interpretable predictions of acute dermal toxicity with multi-view graph pooling and structure remapping

**DOI:** 10.1093/bioinformatics/btae438

**Published:** 2024-07-04

**Authors:** Xinqian Ma, Xiangzheng Fu, Tao Wang, Linlin Zhuo, Quan Zou

**Affiliations:** School of Data Science and Artificial Intelligence, Wenzhou University of Technology, Wenzhou 325027, China; College of Computer Science and Electronic Engineering, Hunan University, Changsha 410012, China; School of Data Science and Artificial Intelligence, Wenzhou University of Technology, Wenzhou 325027, China; School of Data Science and Artificial Intelligence, Wenzhou University of Technology, Wenzhou 325027, China; Institute of Fundamental and Frontier Sciences, University of Electronic Science and Technology of China, Chengdu 611730, China

## Abstract

**Motivation:**

Accurate prediction of acute dermal toxicity (ADT) is essential for the safe and effective development of contact drugs. Currently, graph neural networks, a form of deep learning technology, accurately model the structure of compound molecules, enhancing predictions of their ADT. However, many existing methods emphasize atom-level information transfer and overlook crucial data conveyed by molecular bonds and their interrelationships. Additionally, these methods often generate “equal” node representations across the entire graph, failing to accentuate “important” substructures like functional groups, pharmacophores, and toxicophores, thereby reducing interpretability.

**Results:**

We introduce a novel model, GraphADT, utilizing structure remapping and multi-view graph pooling (MVPool) technologies to accurately predict compound ADT. Initially, our model applies structure remapping to better delineate bonds, transforming “bonds” into new nodes and “bond-atom-bond” interactions into new edges, thereby reconstructing the compound molecular graph. Subsequently, we use MVPool to amalgamate data from various perspectives, minimizing biases inherent to single-view analyses. Following this, the model generates a robust node ranking collaboratively, emphasizing critical nodes or substructures to enhance model interpretability. Lastly, we apply a graph comparison learning strategy to train both the original and structure remapped molecular graphs, deriving the final molecular representation. Experimental results on public datasets indicate that the GraphADT model outperforms existing state-of-the-art models. The GraphADT model has been demonstrated to effectively predict compound ADT, offering potential guidance for the development of contact drugs and related treatments.

**Availability and implementation:**

Our code and data are accessible at: https://github.com/mxqmxqmxq/GraphADT.git.

## 1 Introduction

The skin is the primary pathway for harmful substances to enter the body and a susceptible target organ, making compound-induced acute dermal toxicity (ADT) a significant health risk. Compounds like tetraethyl pyrophosphate (TEPP) and 2,4-dinitrophenol, common in industrial chemicals, pesticides, and cosmetics, illustrate this point. Continuous short-term contact with the skin can lead to adverse effects (BEE and TEST 2017). ADT symptoms include skin redness, swelling, burning, and itching, and may progress to severe chemical burns or necrosis. Furthermore, once absorbed through the skin and into the bloodstream, these chemicals can trigger systemic reactions such as headaches, nausea, and central nervous system damage. In extreme cases, high exposure levels may cause life-threatening conditions like organ failure and severe health issues. These studies underscore the need for timely identification, stringent prevention, control, and swift response to potentially hazardous chemicals. ADT research is included in the “6-pack” suite of tests, aimed at identifying human exposure risks to active ingredients in agrochemicals and related products (Van Der Kamp and Elliott 2021). However, due to animal welfare and ethical concerns, research on mammals is poised to end. Moreover, animal experiments for a single pesticide involve substantial time and financial costs. These factors have collectively spurred the development of computational simulation methods.

The big data era and high-throughput screening technology have led to the generation and collection of massive amounts of ADT experimental data in public databases. These data have fueled the development and validation of computational simulation models, now used for ADT prediction across various fields. For instance, Weltje *et al*. (2017) proposed a computational model that utilizes fish data to predict ADT in terrestrial amphibians from plant protection products, aiming to minimize experimental testing. Concurrently, Luechtefeld *et al*. (2018) introduced the RASAR model, which uses binary fingerprints and Jaccard distance to define chemical similarity and predict potential hazards of related compounds based on data from known hazards. Additionally, Borba *et al*. (2022) gathered extensive data to develop a machine learning-based prediction model for “6-pack” toxicity, including ADT. These studies have amassed valuable data and introduced reference models, significantly advancing toxicity prediction, including for ADT. However, these computational models heavily depend on manually crafted features, which constrains their accuracy and generalizability.

Recently, graph neural network (GNN) technology has rapidly advanced and found widespread application in various biochemical research areas. Common applications include predicting interactions (Wei *et al*. 2023, Ma *et al*. 2024, Zhou *et al*. 2024), molecular properties (Wieder *et al*. 2020), and ADMET properties (Peng *et al*. 2020, Wang *et al*. 2024). GNN excels in analyzing topological structures, which enhances its utility in these domains. Shen *et al*. (2023) developed the Mol-GDL model, which effectively predicts various molecular properties by modeling molecular topology and highlighting the significance of non-covalent interactions. Choo *et al*. (2023) introduced the FinGAT model, which combines 2D fingerprints and graph representations of molecules, demonstrating strong performance in predicting antibiotic activity. Shen *et al*. (2024) proposed a model called CGCN, which incorporates Ollivier-Ricci curvature information and excels in predicting biomolecular interactions. Inspired by these capabilities, recent studies have explored using GNNs to predict compound molecular toxicity. Ketkar *et al*. (2023) evaluated five GNN models across four acute toxicity tasks, highlighting the high accuracy and interpretability of the Attentive FP model (Li *et al*. 2022). Cremer *et al*. (2023) used equivariant GNNs (Han *et al*. 2022), particularly the equivariant Transformer, to predict compound toxicity through a 3D molecular structure representation. Lou *et al*. (2024) combined experimental data from rabbits and rats, using various machine learning and GNN models to predict ADT in these animals. Torres *et al*. (2023) introduced the FS-GCvTR model, a few-shot learning approach utilizing graph neural networks and convolutional Transformer technology. This model effectively integrates local spatial and global information in molecular graph representations, significantly enhancing toxicity and side effect predictions under data-scarce conditions. These studies, which assess toxicity by analyzing compounds’ “overall” structures, demonstrate GNNs’ strong potential in this field. However, compound properties, including toxicity, often rely on “critically important” substructures, a significant limitation in these studies.

Representative GNN-based models excel in ADT prediction tasks, yet they encounter significant challenges. Initially, these models predominantly focus on atom-to-atom (node) information transfer within molecular graphs, often overlooking the valuable data from chemical bonds (edges). Second, ADT prediction fundamentally involves graph classification, necessitating efficient graph representation. Existing GNN models use chemical structures (molecular graphs) for message aggregation and updating, resulting in node representations. This process culminates in a graph representation, achieved through average or max pooling, which is used to predict the compound’s ADT. Clearly, these GNN models emphasize “equal” node representations and fail to differentiate “important” substructures like dominant functional groups or toxicophores. Given the diversity of substructures and functional nodes within a compound, they should be distinctly represented in graph-level models to accurately reflect their roles in pharmacological and toxicological effects. For instance, the nitro (–NO_2_) substructure significantly contributes to a molecule’s toxicity.

To overcome these challenges, we developed the GraphADT model for ADT prediction, which utilizes multi-view graph pooling (MVPool) and structure remapping techniques. The GraphADT model comprises three primary steps: Initially, it maps “bonds” in the molecular graph to new nodes, enhancing bond representation by integrating features of adjacent atoms. A new molecular graph is constructed by mapping “bond-atom-bond” into new edges, thus exposing inter-bond relationships not depicted in the original molecular graph. Second, the model uses MVPool to generate three node importance rankings based on topology and features, utilizing an adaptive attention mechanism to learn the weight of each ranking before integrating them to identify “important” nodes. Finally, both the original and remapped molecular graph representations are contrastively trained to learn the final graph representation for ADT prediction. Overall, the main contributions are summarized as follows:

- This study presents an interpretable model for predicting compound ADT using structure remapping and MVPool technologies, yielding reliable results.
- This study uses structure remapping technology, assigning chemical bonds as nodes and “bond-atom-bond” configurations as edges, to construct a novel molecular graph. This emphasizes bond information and potential inter-bond relationships during message propagation across the graph, uncovering complex molecular patterns and interactions.
- This study leverages MVPool technology to generate diverse views of node importance rankings, integrating adaptive attention mechanisms to refine these rankings and highlight “important” nodes or structures, thus enhancing model interpretability.
- This study conducts contrastive training between the original and remapped molecular graph representations, thus enhancing the compound’s graph representation.

## 2 Materials and methods

**Figure 1.**
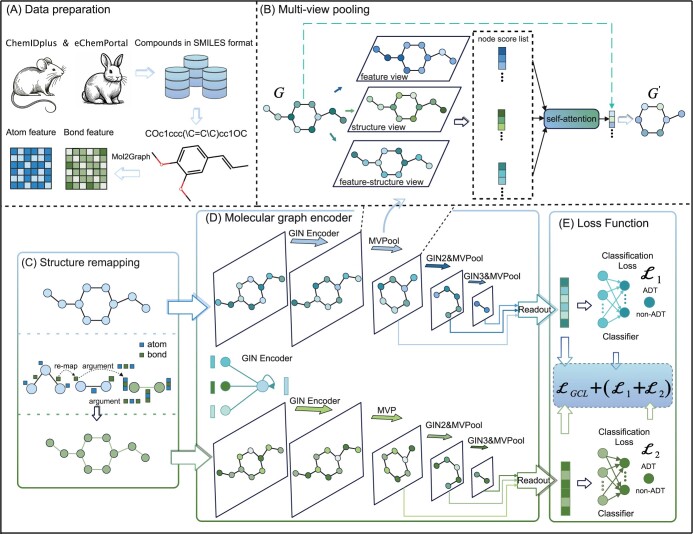
The GraphADT model architecture comprises four main components: (A) data preparation, (C) structure remapping, (D) molecular graph representation extraction, and (E) loss function. Each layer of component (D) includes a GIN encoder and a (B) multi-view graph pooling (MVPool) component. Graph contrastive training is subsequently conducted to determine the molecule’s final representation.

This study develops an innovative model, named GraphADT, which incorporates structure remapping and MVPool techniques to efficiently perform ADT prediction. The architecture diagram of GraphADT is depicted in Figure 1. Initially, chemical bonds are converted into new nodes, and “bond-atom-bond” are transformed into new edges, forming a remapped molecular graph. Subsequently, three node importance rankings are generated based on node structure, features, and a combination of structure and features. The weights of these rankings are automatically learned and integrated via a self-attention mechanism to establish a robust node importance ranking, from which “important” nodes are selected. Finally, the representations of both the original and remapped graphs are contrastively trained to learn the final representation for accurate ADT prediction (as described in Supplementary Sections S7 and S8). The Materials, Problem Formulation, and Model Overview sections are presented in Supplementary Sections S1–S3, respectively.

Our model differs significantly from existing ADT prediction models in two key areas. First, we introduce a structure remapping technique that creates new molecular graphs, enabling message propagation to fully account for potential inter-bond relationships and bond information. Second, our MVPool technique derives node importance rankings, highlighting “important” nodes and structures rather than treating all nodes “equally”.

### 2.1 Structure remapping

Inspired by previous work (as described in Supplementary Section S4), we propose a novel graph representation technology for compounds, termed structure remapping. Initially, input the SMILES notation of the compound and convert it into a directed graph G=<X,E,A>. *X* represents the set of all atom vectors in *G*, with the *u*th atom vector denoted as $X_{u}\in X$. Each atom vector contains the atom symbol, total bond count, formal charge, number of bonded hydrogens, hybridization state, aromatic system status, and atom mass. *E* denotes the set of all chemical bond vectors, with $E_{u,v}\in E$ representing the bond vector from atom *u* to atom *v*. Each bond vector includes the bond type, its conjugation status, and its inclusion in a ring. *A* represents the adjacency matrix of the molecular graph *G*, with $A_{u,v}\in A$ indicating the presence of a bond between atoms *u* and *v*.

The original molecular graph of the compound is structurally remapped to create an enhanced molecular graph, denoted as $G^{r}=(X^{r},E^{r},A^{r})$. In *G^r^*, “bonds” are mapped to nodes, integrating information from directly connected atoms:

(1) $$X_{u,v}^{r}=X_{u}||E_{u,v}||X_{v};\;X_{u},X_{v}\in X,\mathrm{and}\;E_{u,v}\in E$$

where *X^r^* denotes a set of remapped node vectors, and || symbolizes the concatenation operation. And “bond-atom-bond” configurations are mapped as edges, incorporating the edges $E_{u,v}$ and $E_{v,z}$ from the original molecular graph *G* and their connected atom *v*:

(2) $$E_{uv,vz}^{r}=E_{u,v}||X_{v}||E_{v,z};\;E_{u,v},E_{v,z}\in E\;\mathrm{and}\;X_{v}\in X,$$

where *E^r^* represents the set of edge vectors. The adjacency matrix *A^r^* of graph *G^r^* is then defined as:

(3) $$A_{uv,vz}^{r}=1,\;A_{u,v},A_{v,z}\in A,$$

Following these steps and using the molecular SMILES, both the original molecular graph G=<X,E,A> and its structure remapped graph $G^{r}=(X^{r},E^{r},A^{r})$ are constructed.

### 2.2 Molecular graph encoder

The GraphADT model utilizes a multi-layer molecular graph encoder to extract both the representations of compound’s original molecular graph and its remapped graph. The molecular graph encoder comprises three main modules: the Graph Isomorphism Network (GIN) (as described in Supplementary Section S6), MVPool, and a multi-layer attention module. Subsequently, these modules will be described in detail.

### 2.3 Multi-View graph pooling

Building on previous research (Lee *et al*. 2019), we propose a novel MVPool technology designed to address the existing challenges (as described in Supplementary Section S5). The fundamental concept of this technology involves generating multiple node importance rankings from different perspectives and synthesizing these using an adaptive attention mechanism to reduce deviations among views. This approach yields a robust node importance ranking, effectively highlighting critical nodes and structures.

Establishing a node importance ranking is crucial for graph pooling tasks. The concept of “node importance” varies, reflecting diverse interpretations of graph data. In ADT prediction tasks, molecular graphs provide detailed information on structure and node features. Therefore, our objective is to derive node importance rankings based on the node’s structure, features, and the combinations of structure and features, respectively.

#### 2.3.1 Node structure view

The node structure, including its degree and the shortest path between nodes, significantly indicates its importance (Zafarani *et al*. 2014). Due to computational constraints, simpler degree centrality was chosen as the benchmark for scoring node importance. However, directly calculating degree centrality yields higher integer scores. For instance, if a node *u* has five neighbors, its degree score is 5. This scoring scale may not align well with other criteria, potentially leading to significant deviations. Consequently, we adjusted the degree centrality to enhance its compatibility with scoring criteria from other perspectives:

(4) $$p_{c}^{h}=\sigma (\alpha \cdot \log(\deg(A^{h})+\varepsilon )+\beta ),$$

where $p_{c}^{h}$ denotes the importance score of the node in the *h*th layer, *A* is the adjacency matrix of the molecular graph, and the *degree*() function calculates the node’s degree. *ε* is a small positive constant (e.g., e-5) utilized in logarithmic operations. *α* and *β* are learnable parameters designed to automatically adjust the scale of the degree centrality score. *σ* represents the sigmoid function, which normalizes the degree values to a range between 0 and 1.

#### 2.3.2 Node feature view

Besides structure information, nodes often possess rich feature data that significantly reveal their essence. For instance, in a compound’s molecular graph, node features such as atom type, atom symbol, total number of bonds, and formal charge are essential for elucidating the graph’s properties. Consequently, assessing node importance based on their features holds significant potential. A Multilayer Perceptron (MLP) is used to compute the node importance score:

(5) $$p_{f}^{h}=\sigma (\mathrm{MLP}(Z^{h})),$$

where $p_{f}^{h}$ denotes the importance score of the node in the *h*th layer, and *Z^h^* represents the node’s embedding in the same layer. The output dimension of the MLP function is configured to 1.

#### 2.3.3 Node structure-feature view

To comprehensively assess node importance, integrating node structure and feature is a logical approach. Consequently, we utilize an advanced version of the PageRank method (Page *et al*. 1999, Haveliwala 2002) to estimate the importance of each node in the graph. Specifically, a GNN maps each node to a value between [0,1], reflecting its structure and attribute data. These values are then iteratively propagated using the advanced PageRank method until convergence:

(6) $$PR=\sigma ({\hat{D}}^{-\frac{1}{2}}{\hat{A}}^{k}{\hat{D}}^{-\frac{1}{2}}Z^{h}\Phi ),$$

where $\hat{A}=A+I$, *I* represents the identity matrix, $\hat{D}$ is the diagonal matrix of $\hat{A}$. And Φ denotes a learnable weight.

(7) $$p_{cf}^{h}=\sigma (\delta {\left(I-(1-\delta ){\hat{A}}^{h}\right)}^{-1}PR),$$

where $p_{cf}^{h}$ denotes the node’s importance score in the *h*th layer, is the restart probability, constrained to (0, 1]. Direct calculation of Equation (7) can be computationally intensive due to the inverse operation; instead, the power iteration method may be used to approximate the PageRank value (Gasteiger *et al*. 2018).

#### 2.3.4 Adaptive attention mechanism

Given that a single perspective may result in biased evaluations, significant differences between various views are common. Integrating various importance ranking views can mitigate deviations, enhance inter-view cooperation, and thus stabilize node importance rankings. To accomplish this, we have designed an adaptive attention mechanism that automatically learns the weight of each view. Building on existing research on attention mechanisms (Bahdanau *et al*. 2015), the weight of view *j* is defined as follows:

(8) $$g_{j}=\frac{\;\text{exp}(\sigma (p_{\mathrm{all}}^{T}w_{j}+b_{j}))}{\sum_{i=1}^{3}exp(\sigma (p_{\mathrm{all}}^{T}w_{i}+b_{i}))},$$

where *p_all_* represents the concatenated vector of all views, with *w_j_* and *b_j_* as parameters of view *j*. Consequently, a high *g_j_* value signifies the importance of view *j*. Based on this, the final node importance score is calculated as follows:

(9) $$p=\sum_{j=1}^{3}g_{j}\cdot p_{j}.$$

Nodes retained by the pooling operation are then selected based on these scores. Specifically, nodes are ranked by their importance scores, and the top-ranked nodes are retained:

(10) $$\mathrm{ind}=\mathrm{top}-\mathrm{rank}(p,\left\lceil r\cdot N^{k}\right\rceil ),\;\hat{p}=\mathrm{sigmoid}(p(\mathrm{ind}));$$

(11) $$Z^{h+1}=Z^{h}(\mathrm{ind},:)⊙({\hat{p}}^{T}1),\;A^{h+1}=A^{h}(\mathrm{ind},\mathrm{ind}),$$

where *r* denotes the pooling ratio. The top-rank function yields indices for the top $\left\lceil r\cdot N^{k}\right\rceil$ nodes. $Z^{h}(\mathrm{ind},:)$ represents the row vector of node *ind* in the graph, used to construct the node representation matrix $Z^{h+1}$ of the *h *+* *1 layer. $A^{h}(\mathrm{ind},\mathrm{ind})$ involves extracting the relevant rows or columns to construct the adjacency matrix $A^{h+1}$ for the *h *+* *1 layer. Here, **1** represents a matrix entirely composed of ones. The multiplicative gating operation in $\hat{p}$ facilitates end-to-end backpropagation within the model. Each encoder layer comprises a GNN encoding layer and a multi-view pooling layer, iterating *K* times and integrating outputs across these layers via a multi-layer attention mechanism.

## 3 Results

This section comprises: (1) clarification of experimental settings (as described in Supplementary Section S9); (2) comparative benchmark testing to evaluate the GraphADT model’s performance; (3) ablation studies to ascertain the importance of GraphADT model’s core modules; (4) multiple case analyses to assess the model’s interpretability.

**Figure 2.**
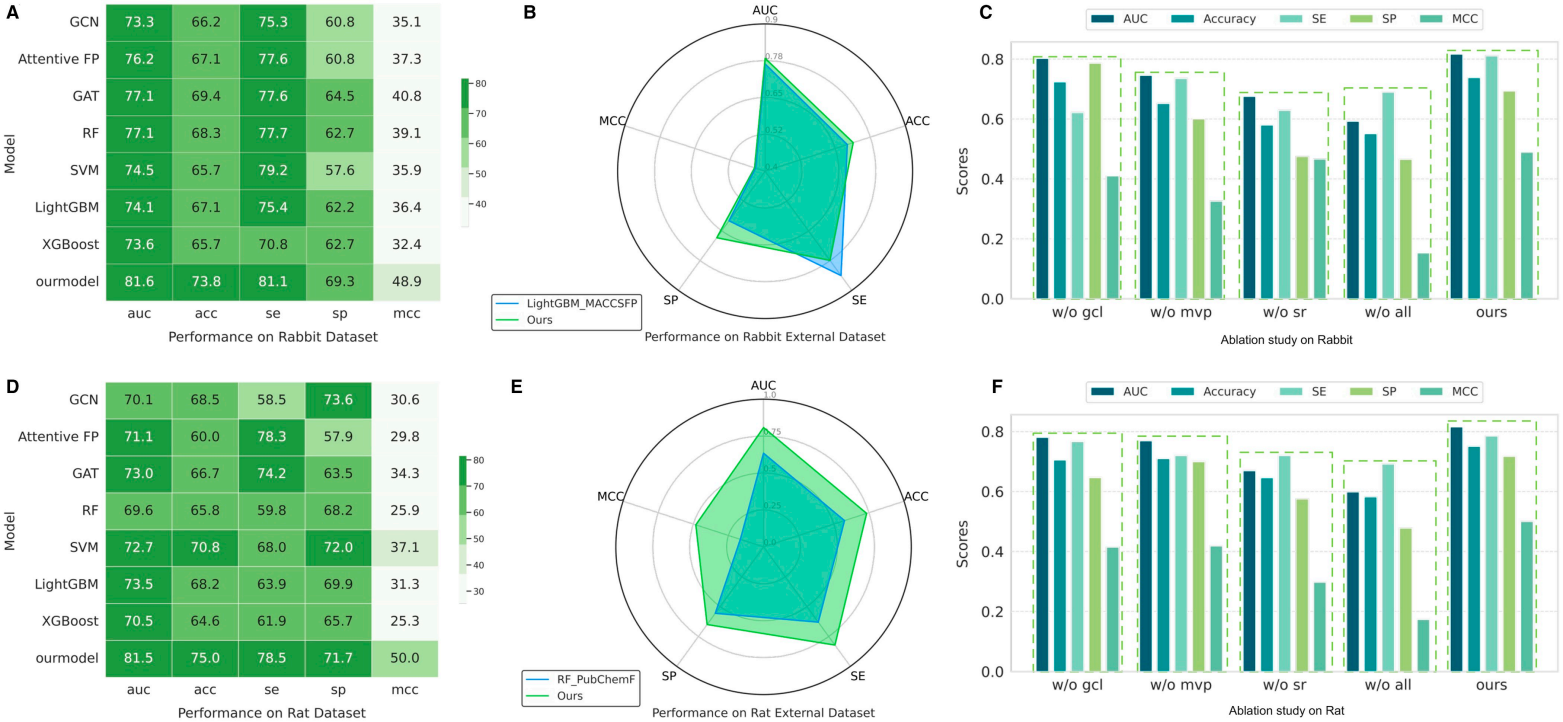
Performance comparison. Results of the proposed GraphADT model and seven baseline models on the (A) Rabbit and (D) Rat test sets. Results of the proposed GraphADT model and the best-performing comparison model on the (B) Rabbit and (E) Rat external datasets. Results of ablation experiment on the (C) Rabbit and (F) Rat test sets.

Figure 2B and E compare performance on external datasets, while Fig. 2C and F present ablation study results. The Key Substructure Overview, Comparison Models, Evaluation on External Datasets, Ablation Experiment, Parameter Experiment, Molecular Graph Representation Analysis, and Molecular Skeleton Alignment Analysis sections are presented in Supplementary Sections 10–16, respectively.

### 3.1 Performance comparison

Figure 2A presents the results of all models on the Rabbit test set. The GraphADT model demonstrated superior performance, achieving an AUC of 81.6%, ACC of 73.8%, MCC of 48.9%, SE of 81.1%, and SP of 69.3%. These metrics surpassed the second-best method by 4.5%, 4.4%, 8.1%, 3.5%, and 4.8%, respectively. Figure 2D displays the performance of all models on the Rat test set. The GraphADT model again outperformed others, recording an AUC of 81.5%, ACC of 75.0%, MCC of 50.0%, and SE of 78.5%. These results confirm that the GraphADT model significantly surpasses all comparative models in predicting the potential ADT of compounds. This superior performance may be attributed to two factors: First, the model uses structure remapping technology to represent “bonds” as nodes and “bond-atom-bond” configurations as edges, focusing on bond information and potential inter-bond relationships. Second, it utilizes MVPool technology to generate various node importance ranking views. An adaptive attention mechanism integrates these views, enhancing inter-view collaboration and minimizing single-view deviations. Consequently, a robust node importance ranking emerges, highlighting “important” nodes and structures.

### 3.2 Chemical structure-based interpretability analysis

We used the EdgeSHAPer method (Mastropietro *et al*. 2022) for compound structure analysis. In the structure remapped graph of a compound, nodes represent “bonds”. Consequently, we calculated the Shapley value for each node. In this study, the Shapley value quantified the average marginal contribution of each “bond” to the predicted ADT by the model. Explanatory analysis was conducted on 133 positive samples from the Rabbit test set and 132 from the Rat test set, respectively.

**Figure 3.**
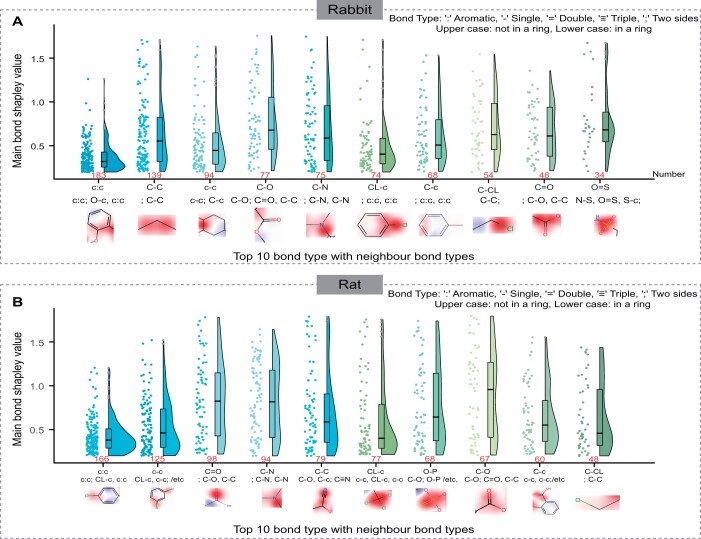
Results of interpretability analysis of GraphADT model predictions on the (A) Rabbit and (B) Rat test sets.

A Shapley value threshold of 0.2 was established, and the ten most critical chemical bonds and their adjacent bonds were selected based on their frequency of occurrence. The occurrences of these chemical bonds and their adjacent bonds were counted, with results displayed in Fig. 3. Based on the Shapley values, key chemical bonds potentially triggering ADT in the Rabbit dataset were identified. Figure 3A lists the top 10 functional groups identified: phenol, linear propyl, cyclic structures, ethers, esters, tertiary amines, chlorobenzene, alkylbenzene, chloroalkyl, and ester groups. When the primary bond Shapley value exceeds 0.2, the contributions of these functional groups are approximately 9.8%, 6.4%, 5.3%, 12.9%, 9.3%, 31.0%, 7.3%, 24.0%, 20.8%, and 11.7%, respectively. Notably, the ether structure demonstrated a high Shapley value in the model’s predictions.

Using the Shapley value, we identified key chemical bonds that may trigger ADT in the Rat dataset. Figure 3B lists the top 10 functional groups, which include chlorobenzene, *O*-dichlorobenzene, acyl, cyano, acetoxy, trichloromethyl, phosphoryl, ester, aromatic carboxylic acid, and halogenated hydrocarbon. Their contributions are 11.4%, 4.8%, 15.3%, 8.5%, 3.8%, 7.8%, 10.3%, 14.9%, 6.6%, and 27.1%, respectively. Observations indicate that the first five functional groups significantly contribute to toxicity, collectively accounting for approximately 43.8%. Notably, the ester group exhibits a higher Shapley value.

We analyzed potential factors influencing model performance and investigated key atoms or substructures that determine a compound’s ADT. This analysis aims to enhance the interpretability of model prediction results. This analysis is intended to elucidate the causes of compound ADT and guide the design and improvement of contact drugs.

## 4 Conclusion

We initially reviewed existing compound ADT prediction models and identified their challenges. For instance, machine learning-based methods primarily focus on features like the SMILES sequence of compounds, often overlooking the molecule’s chemical structure. GNN-based models can effectively learn a compound’s structure to perform ADT prediction. However, these methods typically concentrate solely on atom-level message passing, neglecting crucial information conveyed by chemical bonds and their potential interrelationships. Additionally, most methods treat nodes as “equal,” failing to emphasize important nodes or structures, thus lacking interpretability. Consequently, we propose a novel compound ADT prediction model utilizing structure remapping and MVPool technologies. Initially, structure remapping technology is introduced to map “bonds” to nodes and “bond-atom-bond” configurations to edges. This approach aims to maximize attention to the crucial information carried by chemical bonds and their potential relationships during message propagation. Second, MVPool technology is introduced to amalgamate information from various perspectives, reducing deviations inherent in single-view analyses. Subsequently, a robust node ranking system is developed collaboratively to highlight important nodes or substructures, significantly enhancing the model’s interpretability. Finally, a graph comparison learning strategy is used to train both the original and structure remapped molecular graphs, improving the representation. Collectively, experiment results confirm that the GraphADT model effectively predicts the potential ADT of compounds and may guide the development of contact drugs and their treatments.

Theoretically, the GraphADT model is adaptable to tasks involving molecular classification and regression based on topological structures. The model processes inputs of physical properties and chemical structure information of compounds, making it suitable for tasks like predicting compound toxicity and metabolic stability. Consequently, the GraphADT model holds potential for application in these areas. However, the GraphADT model has limitations; it solely extracts features from molecular chemical structures and does not utilize multi-source data including sequences, images, 3D geometries, and textual descriptions. Additionally, being trained solely on a specific dataset hinders the model from acquiring general knowledge about molecular representations, thus limiting its generalization capabilities. In future work, we aim to integrate multi-source data to enhance molecular representations and use pre-training or large-model technologies to assimilate general molecular knowledge, thereby improving the model’s generalization capabilities.

## Supplementary Material

btae438_Supplementary_Data
