# Supplementary material for: GraphADT: empowering interpretable predictions of acute dermal toxicity with multi-view graph pooling and structure remapping
Source: Bioinformatics. 2024 Jul 4;40(7):btae438. doi: 10.1093/bioinformatics/btae438 (PMC11245318; doi:10.1093/bioinformatics/btae438)
Supplement: btae438_Supplementary_Data [file btae438_supplementary_data.pdf]

1 Materials

In this study, the ADT datasets were sourced from prior research [Lou et al., 2024]. The datasets, comprising compound ADT data, were collated from two databases, ChemIDplus [Tomasulo, 2002] and eChemPortal[1], followed by preliminary screening. According to the Globally Harmonized System (GHS) [CHEMICALS, 2001], compounds are classified into five categories based on their LD50 values— the single dose required to kill 50% of a test animals. Compounds in the first four categories are considered to exhibit ADT, while those in the fifth category and those not meeting GHS standards are classified as non-ADT. Additionally, two compounds incorrectly represented in the graph structure were removed. Ultimately, data from 1734 rabbits and 1678 mice were collected. Additionally, two external test sets were obtained from the NITE database[2] and underwent similar processing. The dataset was divided into training and test sets at a ratio of 4:1. Given the imbalance between toxic and non-toxic compounds, undersampling was applied to the training set, yielding 1030 rabbit and 902 rat samples. It is important to note that the test set was not subjected to sampling.

Table S1: Results of ablation experiment.

		Rabbit			Rat	
datasets	training	test	external	training	test	external
ADT	515	130	124	451	97	73
non-ADT	873	217	212	892	239	196
Total	1388	347	336	1343	336	269

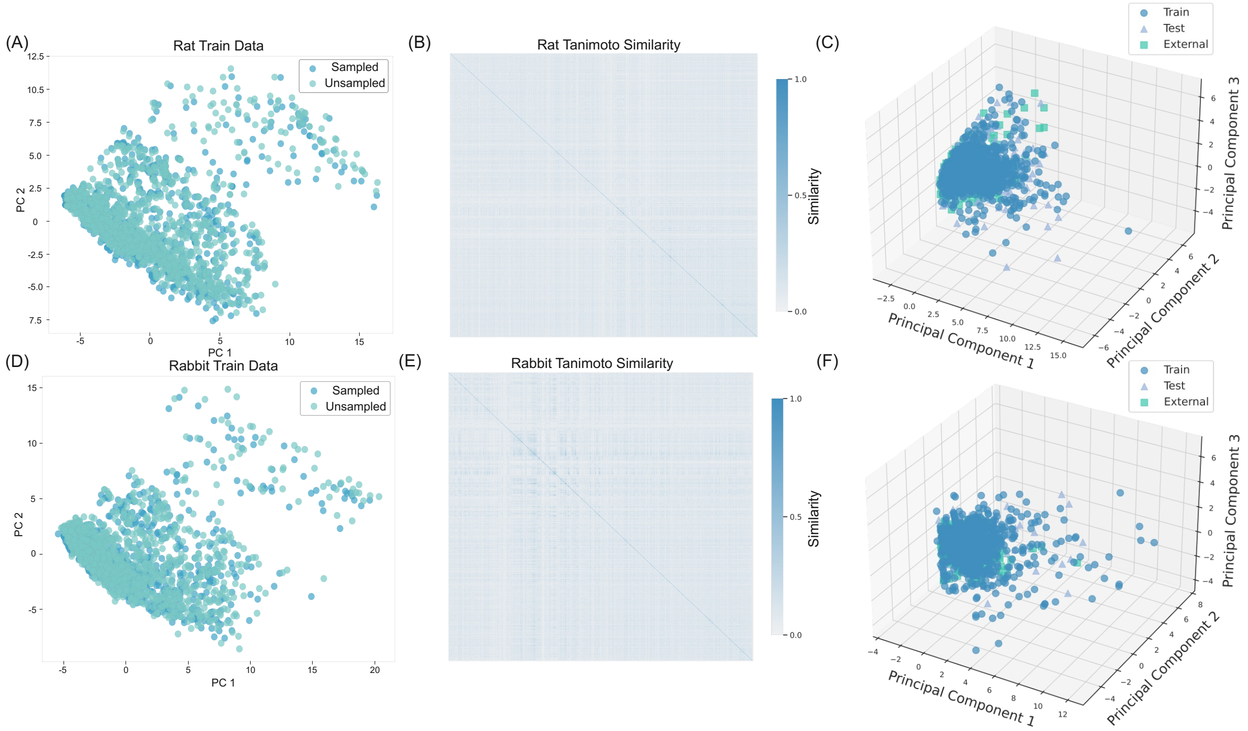

Figure S1: (A) Rat and (D) rabbit train data sampling diagram. Tanimoto similarity heat map of (B) Rat and (E) Rabbit datasets. Spatial distribution of (C) Rat and (F) Rabbit datasets (training set, test set and external validation set).

Principal component analysis (PCA)[3] was used to visualize the training set before and after undersampling, as illustrated in Figures S1(A) and (D). The results indicate an even distribution of undersampled data, suggesting an improvement in training data quality. Additionally, analyzing the Murcko framework [?] of compounds and calculating Tanimoto similarity[4] elucidates the diversity of chemical structures within the dataset. Murcko framework analysis involves exploring chemical differences in the ADT dataset by removing all substituents, retaining only the unique ligand and ring structures. The analysis revealed 561 and 354 unique Murcko frameworks

[1] https://www.echemportal.org/echemportal/
[2] https://www.nite.go.jp/en/chem/ chrip/chrip_search/systemTop
[3] https://en.wikipedia.org/wiki/Principal_component_analysis
[4] https://en.wikipedia.org/wiki/Jaccard_index

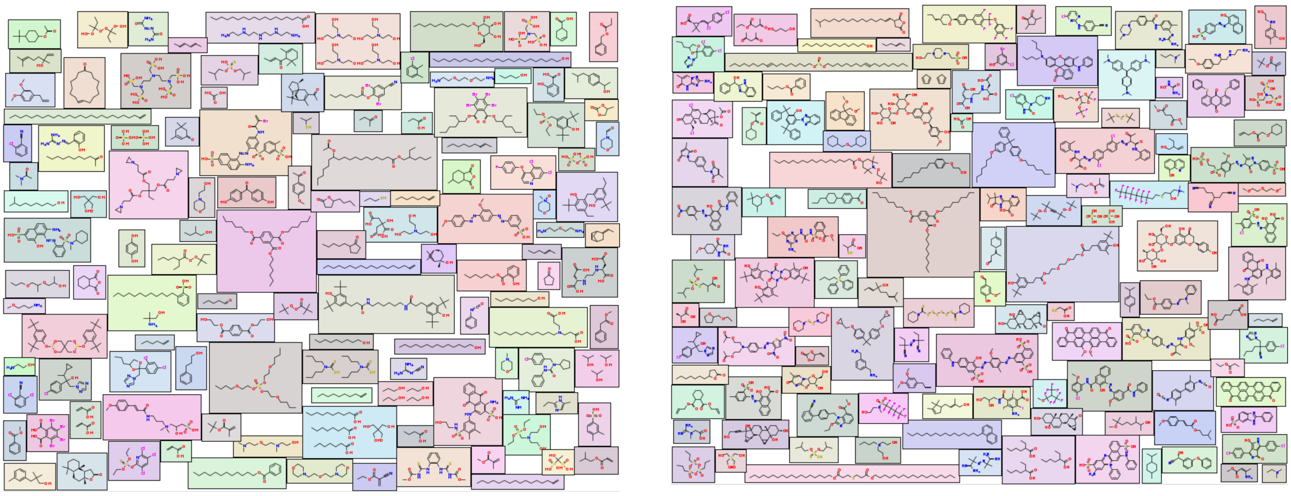

(A) (B)

Figure S2: Cloud visualization of (A) Rabbit and (B) Rat datasets.

in the Rat and Rabbit ADT datasets, respectively. In both datasets, over 85% of the frameworks consisted of just one or two molecules. Concurrently, the average Tanimoto similarity for all molecules was calculated using ECFP4 fingerprints for both Rat and Rabbit datasets, yielding values of 0.082 and 0.085, respectively, as depicted in Figures S1(B) and (E). The aforementioned analysis confirms that the dataset utilized for performance evaluation possesses adequate compound diversity.

Additionally, PCA was employed to display the training, test, and external validation sets on a three-dimensional scatter plot using RDKit descriptors, as shown in Figures S1 (C) and (F). The results indicate a relatively consistent distribution across the three data sets within the chemical space. The statistics for the training, test, and external validation sets are detailed in Table S1. We also conducted statistical analyses of key functional groups and substructures. Based on prior research [Ertl and Rohde, 2012], we enhanced the dataset's visualization, as depicted in Figure S2.

2 Problem Formulation

ADT prediction is modeled as a graph classification task. Assume that the compound set $D = \{D_i | i \in \{1, \cdots, N\}\}$ and its label set $y = \{y_i | y_i \in \{0, 1\}, i \in \{1, \cdots, N\}\}$. Here, $y_i = 1$ indicates that the i-th compound exhibits ADT; $y_i = 0$ indicates the absence of ADT. The primary objective is to train a graph classifier, denoted as f_Θ, to compute scores $\hat{y}$ that determine the ADT status of these compounds. In this model, Θ represents the parameters of the classifier.

3 Model Overview

Figure S3 illustrates the architecture of the GraphADT model, comprising five key components. Component (A) involves collecting ADT data from prior research [Lou et al., 2024], constructing a molecular graph from SMILES notations, and extracting features of atoms (nodes) and bonds (edges). Component (B) generates three node importance rankings based on node structure, feature, structure-feature, respectively, using an adaptive attention mechanism to synthesize these views into a consolidated ranking and identify key nodes. Component (C) maps "bonds" to new nodes and "bond-atom-bond" to new edges, creating an enriched molecular graph that integrates features of adjacent atoms to enhance the representation of remapped nodes and edges. Component (D) employs the graph isomorphism network (GIN) [Xu et al., 2018] and component (B) to extract representations of both the original and structure remapped molecular graphs, integrating these using a hierarchical attention mechanism. Component (E) contrastively trains the representations from the original and remapped molecular graphs, derived in component (D), to establish the final molecular representation and predict its ADT. The following sections will detail the functions of these components.

4 Inspiration of Structure Remapping

In graph theory, the concept of transforming edges of an original graph into nodes to construct a line graph was first introduced by Harary and Norman in 1960 [Harary and Norman, 1960]. A significant advantage of the line

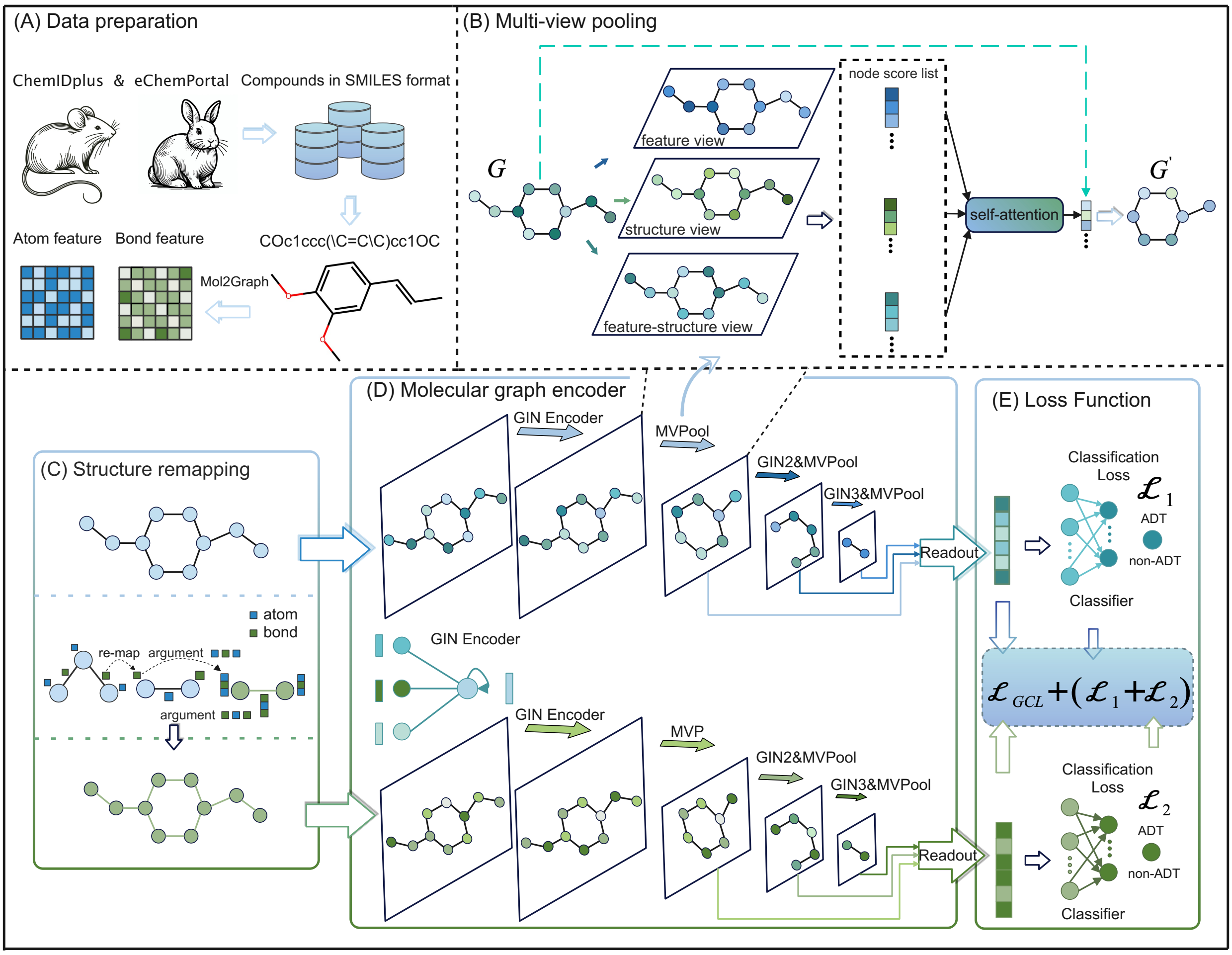

Figure S3: The GraphADT model architecture comprises four main components: (A) Data preparation, (C) Structure remapping, (D) Molecular graph representation extraction, and (E) Loss function. Each layer of Component (D) includes a GIN encoder and a (B) Multi-View graph pooling (MVPool) component. Graph contrastive training is subsequently conducted to determine the molecule's final representation.

graph is its ability to highlight the information contained in edges and the potential relationships between these edges during information transmission. Within a compound's structure, the "bond" is a crucial component influencing key structures, including functional groups, pharmacophores, toxicophores, and binding pockets. In current graph classification tasks, such as ADT prediction, the emphasis is primarily on message transfer between atoms, often overlooking the bond information and potential inter-bond relationships. This oversight can result in biased interpretations of compound structures by the respective models. Using a line graph for molecular representation can intuitively enhance the bond information and uncover potential inter-bond relationships.

5 Inspiration of Multi-View Graph Pooling

Following node representation extraction using the GNN model, the existing ADT prediction model integrates these representations into a unified graph representation via average or maximum pooling. These models consider each node "equally", neglecting to emphasize "important" substructures like dominant functional groups or toxicophores. Given the diversity of substructures and functional nodes within a compound's structure, it is crucial to differentiate these elements in the graph-level representation to accurately reflect their pharmacological and toxicological impacts. For instance, the nitro (-NO2) substructure significantly contributes to a molecule's toxicity. Graph pooling techniques like SAGpool [Lee et al., 2019] and Diffpool [Ying et al., 2018] have significantly enhanced performance in graph classification tasks. However, these methods assess node importance from a single perspective, potentially introducing bias.

6 GIN Encoder

Compared to commonly used GNNs like GCN, GraphSAGE, and GAT, GIN excels in parsing graph structure information. Consequently, the GIN model was selected for the GraphADT model to extract features from both the original and structure remapped molecular graphs. For graphs $G =< X, E, A >$ and $G^r = (X^r, E^r, A^r)$, GIN executes aggregation and update operations within the nodes' local neighborhoods:

$$z_u^h = MLP((1 + \tau^h) \cdot z_u^{h-1} + \sum_{v \in N(u)} z_v^{h-1}), \tag{1}$$

where z_u^h represents the embedding of node u in the k-th layer of the GIN, τ is the weight parameter, and $N(u)$ denotes the set of adjacent nodes to node u. Subsequently, z_u^h is input into the multi-view graph pooling layer.

7 Graph Contrastive Learning

Graph contrastive learning, an unsupervised learning strategy, enhances the similarity between different views of graph data to improve node representation. In the proposed GraphADT model, molecular graphs are constructed from two perspectives: original molecular graphs and structure remapped graphs. These two views offer complementary insights; the molecular graph focuses on inter-atom interactions, whereas the structure remapped view highlights inter-bond relationships. The graph contrastive learning strategy compels the model to integrate both types of information, thereby refining the molecule's graph representation.

Assuming the training dataset contains N compounds, molecular maps and structure remapped graphs are constructed for each based on its SMILES sequence. For a given compound u, z_u and z_u^r represent its original molecular graph and structure remapped graph, respectively. The loss is calculated using the InfoNCE function [He et al., 2020]:

$$\mathcal{L}_{GCL} = -\frac{1}{N} \sum_{u=1}^{N} log \frac{e^{z_u \cdot z_u^r / \tau}}{\sum_{v=1}^{N} e^{z_u \cdot z_v^r / \tau}} \tag{2}$$

8 ADT Predictor

The optimization objectives for the proposed GraphADT model encompass classification loss and contrastive learning loss. The graph representation of the molecule, derived from both the molecular graph and the structure remapped graph, allows for separate predictions of the final ADT score, with classification loss computed using the BCE function:

$$\mathcal{L}_1 = -\sum_{u=1}^{N} y_u \cdot log\sigma(\hat{y}_u) + (1 - y_u) \cdot log\sigma(1 - \hat{y}_u) \tag{3}$$

$$\mathcal{L}_2 = -\sum_{u=1}^{N} y_u \cdot log\sigma(\hat{y}_u^r) + (1 - y_u) \cdot log\sigma(1 - \hat{y}_u^r) \tag{4}$$

where $\mathcal{L}_o$ denotes the classification loss derived from the molecular graph, and $\mathcal{L}_r$ signifies the classification loss from the structure remapped graph. N denotes the total number of molecules, while σ symbolizes the sigmoid function. For the u-th molecule, $\hat{y}_u$ indicates the predicted ADT score from the molecular map, $\hat{y}_u^r$ from the structure remapped graph, and y_u denotes its true label. The model integrates classification loss with contrastive learning loss as follows:

$$\mathcal{L} = \mathcal{L}_1 + \mathcal{L}_2 + \lambda \mathcal{L}_{GCL}, \tag{5}$$

where λ serves as an adjustable parameter.

During inference, we extract the structure remapped graph representation of the compound to predict its ADT. Unlike the training process, where the optimization objectives include classification loss $\mathcal{L}_1$ from the original molecular graph, classification loss $\mathcal{L}_2$ from the structure remapped graph, and graph contrast learning loss $\mathcal{L}_{GCL}$ (as shown in Figure S3 of the manuscript).

In the structure remapped graph, node information includes properties of two adjacent atoms and their bonds, incorporating bond interactions. This allows the GIN encoder to simultaneously absorb atom and bond information during message propagation, enhancing node representation. In the original molecular graph, node information solely comprises atom properties. Consequently, we utilize the structure remapped graph encoding module to extract molecular representations for ADT prediction.

9 Experiments Setup

The proposed GraphADT model was implemented in a Python and PyTorch Geometric (PYG) environment. The GIN encoder and multi-view graph pooling each consist of three layers. The batch size was set at 256, with 300 training epochs, a learning rate of 0.001, and ADAM chosen as the optimization strategy. The model's predictive architecture comprises three levels: an input layer with 512 dimensions, a hidden layer with 256 dimensions, and an output layer with 1 dimension. The data for model training were randomly split into training and test sets at a 4:1 ratio. Throughout the training process, 10-fold cross-validation was employed to select optimal parameters. The model's performance was comprehensively evaluated using five indicators: area under the ROC curve (AUC), accuracy (ACC), sensitivity (SE), specificity (SP), and Matthews correlation coefficient (MCC).

10 Key Substructure Overview

We provide a concise overview of the key chemical substructures involved in the "Chemical Structure-based Interpretability Analysis" section to support our analysis and conclusions. Recognizing the importance of this foundational work, we aim to strengthen the scientific integrity and reliability of our analytical experiments. Below, we detail the research foundations pertinent to these key substructures.

1. Phenol: Phenolic compounds have extensive industrial applications. Phenol exposure can lead to skin inflammation, contact dermatitis, and pigment loss. Research indicates that local exposure to phenol generates free radicals, triggers oxidative stress, depletes skin antioxidants, and results in skin toxicity [Murray et al., 2007].

2. Chlorobenzene: Chlorobenzene and its derivatives exhibit pronounced ADT, particularly at elevated concentrations and with extensive chlorine substitution. Its toxicity primarily involves direct cytotoxicity and an oxidative stress response [Feltens et al., 2010].

3. Alkylbenzene: Research on guinea pigs demonstrates that prolonged exposure to linear alkylbenzene sulfonates (LAS) can induce skin hyperkeratosis, mononuclear cell infiltration, and skin redness and swelling. High-dose exposure may lead to severe skin necrosis and hair loss [Mathur et al., 2000].

4. Ester group: A comparative study on 14 phthalates revealed that low molecular weight phthalates, like dimethyl and diethyl, exhibit higher acute toxicity to aquatic organisms than their high molecular weight counterparts [Adams et al., 1995].

5. Orthodichlorobenzene: Research indicates that orthodichlorobenzene can induce skin inflammation and contact dermatitis during industrial applications. It is commonly employed in wood preservatives, where it poses considerable irritant and toxic effects on the skin [Downing, 1939].

6. Cyanide group: A study on the acute toxicity of cyanides like hydrogen cyanide, sodium cyanide, and potassium cyanide showed that the LD50 for intact skin was 0.299 mmol/kg. For damaged skin, it significantly decreased to 0.087 mmol/kg. Additionally, the LD50 for sodium cyanide on dry, intact skin was 0.243 mmol/kg, while on wet, intact skin, it was 0.151 mmol/kg [Ballantyne, 1994].

7. Trichloromethyl: Compounds like trichloroethylene under the trichloromethyl group are known to cause severe acute skin reactions, including irritation, erythema, edema, hypersensitivity, and inflammation [Shen et al., 2008].

8. Phosphoryl: Phosphoryl compounds induce acute toxicity, manifesting as skin irritation and systemic poisoning through the inhibition of crucial enzymes like acetylcholinesterase. It is vital to implement protective measures during their handling and use to mitigate exposure risks and health hazards [Hoppe et al., 2008].

9. Aromatic carboxylic acids: At high concentrations or prolonged exposure, aromatic carboxylic acids can cause ADT, characterized by irritation and developmental abnormalities. It is essential to adopt protective measures to minimize exposure risks and health hazards during their handling and use [Dogra et al., 2018].

10. Halogenated hydrocarbons: Halogenated hydrocarbons exhibit marked ADT, featuring irritation, cellular damage, and tumor-promoting effects, due to solvent and metabolite actions. Precautions are imperative when handling and using these compounds to minimize exposure risks and health hazards [Berger and Sozeri, 1987].

11 Comparison Models

In this study, we evaluated the performance of the proposed GraphADT model against seven benchmark models. The benchmarks included Random Forest (RF) [Rigatti, 2017], Support Vector Machine (SVM) [Cortes and Vapnik, 1995], Extreme Gradient Boosting (XGBoost) [Sheridan et al., 2016], Lightweight Gradient Boosting Machine (LightGBM) [Ke et al., 2017], Graph Convolution Network (GCN) [Kipf and Welling, 2016], Graph Attention Network (GAT) [Veličković et al., 2018], and Attention FP [Li et al., 2022], each configured to predict ADT.

12 Evaluation on External Datasets

Additionally, we assessed the performance of the proposed GraphADT model alongside the current best-performing LightGBM_MACCSFP and RF_PubChemFP models on Rat and Rabbit external datasets. The LightGBM_MACCSFP and RF_PubChemFP models were trained using MACCS and PubChem fingerprint data, respectively. To ensure fair comparisons, identical training and test set samples were utilized across all experiments. The model's performance in predicting compound ADT was evaluated using AUC, accuracy, sensitivity (SE), specificity (SP), and Matthews correlation coefficient (MCC), with results presented in Figures S4(B) and (E).

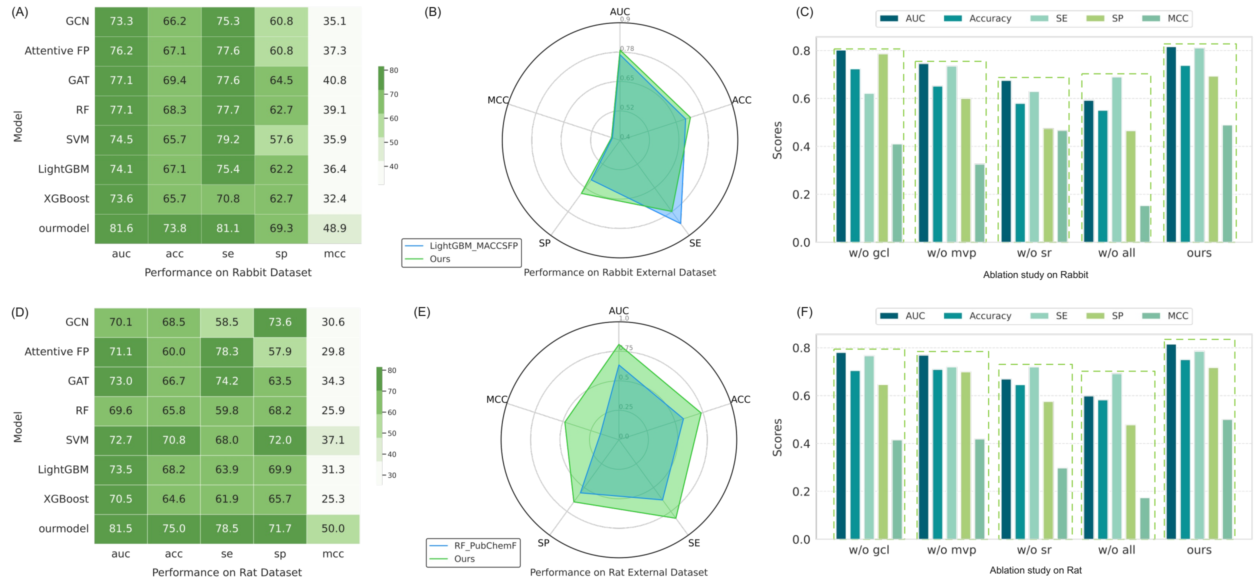

Figure S4: Performance comparison. Results of the proposed GraphADT model and seven baseline models on the (A) Rabbit and (D) Rat test sets. Results of the proposed GraphADT model and the best-performing comparison model on the (B) Rabbit and (E) Rat external datasets. Results of ablation experiment on the (C) Rabbit and (F) Rat test sets.

The GraphADT model demonstrated superior performance across nearly all metrics compared to the LightGBM_MACCSFP and RF_PubChemFP models. In the Rat external dataset, the GraphADT model surpassed the second-best method by margins of 16.6% in AUC, 15.8% in ACC, 31.7% in MCC, 19.2% in SE, and 9.4% in SP. In the Rabbit external dataset, the GraphADT model outperformed the second-best approach by 2.0% in AUC, 2.1% in ACC, 7.1% in SP, and 0.4% in MCC. It only slightly lagged behind in the SE metric.

Specifically, the GraphADT model's AUC metric showed a 16.6% enhancement on the Rat dataset and a 2.0% increase on the Rabbit dataset. This indicates that the GraphADT model effectively discriminates between positive and negative samples. Additionally, the GraphADT model's ACC metric improved by 15.8% on the Rat dataset and by 2.1% on the Rabbit dataset. This demonstrates the GraphADT model's enhanced overall prediction accuracy. The SE metric for the GraphADT model showed a 19.2% improvement on the Rat dataset, though it was marginally lower on the Rabbit dataset. This suggests that the GraphADT model can more effectively identify positive samples, namely toxic compounds. For SP metric, the GraphADT model recorded improvements of 9.4% and 7.1% on the Rat and Rabbit datasets, respectively. This enhancement enables the model to more accurately identify negative (non-toxic) samples, ensuring reliable exclusion of such compounds. The GraphADT model's MCC metric improved by 31.7% on the Rat dataset and by 0.4% on the Rabbit dataset. This confirms the GraphADT model's enhanced and reliable predictive performance. In summary, the GraphADT model demonstrated notable enhancements across various key performance metrics,

affirming its effectiveness and robustness across diverse datasets. Future efforts will focus on refining feature extraction and data augmentation techniques to enhance the model's SE and SP performance further.

13 Ablation Experiment

The proposed GraphADT model comprises two core components: structure remapped, multi-view graph pooling, and a trivial component: graph contrastive learning. To assess the contributions of these components to model performance, we conducted ablation studies on Rat and Rabbit datasets, with results presented in Figures S4(C) and (F). In these experiments, "w/o gcl" indicates removal of the graph contrast learning component, "w/o sr" the removal of the structure remapped component, and "w/o mvp" the omission of the multi-view graph pooling. "w/o all" signifies that all specialized components were removed, leaving only the GIN model for predicting compound ADT. Results indicate that incorporating structure remapped, multi-view graph pooling, and graph comparison learning significantly enhances model performance. Notably, removing either the structure remapped or multi-view graph pooling components significantly degraded model performance, underscoring their critical roles. This emphasizes the importance of focusing on the information conveyed by "bonds" and potential inter-bond interactions. Furthermore, emphasizing "important" nodes or structures significantly optimizes model performance.

14 Parameter Experiment

14.1 Impact of Different GIN Layers

We conducted a five-fold cross-validation experiment on both Rabbit and Rat datasets to assess the impact of varying GIN layer counts on model performance. Results are presented in Figure S5. It is evident that varying the number of GIN layers influences model performance. The effect is relatively minor in the Rabbit dataset but more pronounced in the Rat dataset. Overall, setting the number of GIN layers to 3 yields optimal model performance.

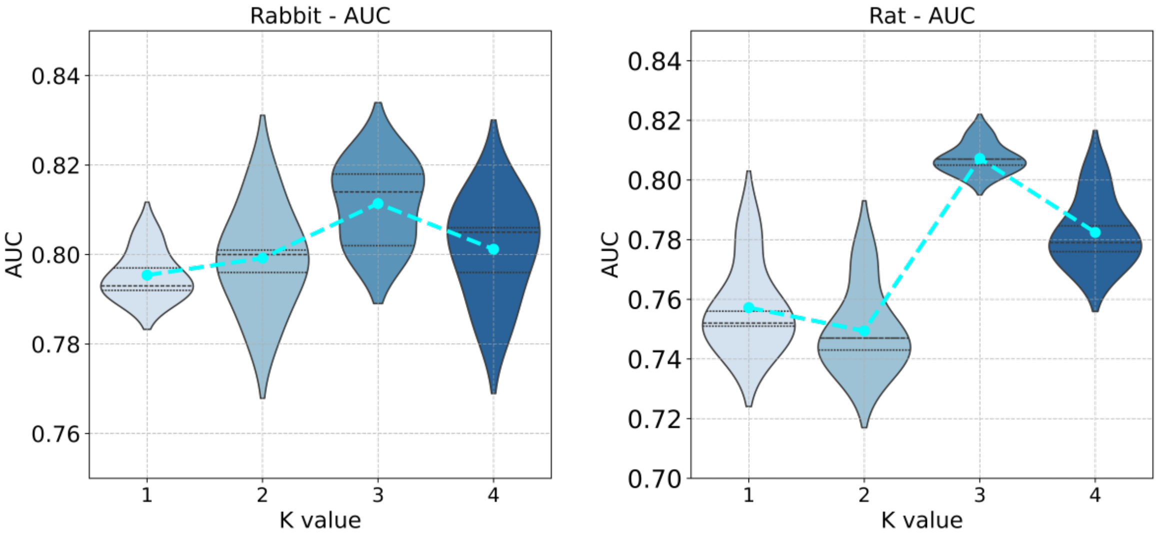

Figure S5: Model AUC across various GIN layer configurations.

14.2 Impact of Different Pooling Strategies

We conducted 10-fold cross-validation experiments on Rabbit and Rat datasets to assess the model's performance with SAGpool, TopKpool, and our newly designed MVPool, respectively. Results are displayed in Figure S6. The model demonstrated comparable performance with both SAGpool and TopKpool. Notably, the model achieved optimal results across all metrics with the MVPool strategy. This demonstrates MVPool's superiority and suggests its potential to discern the compound's key structures.

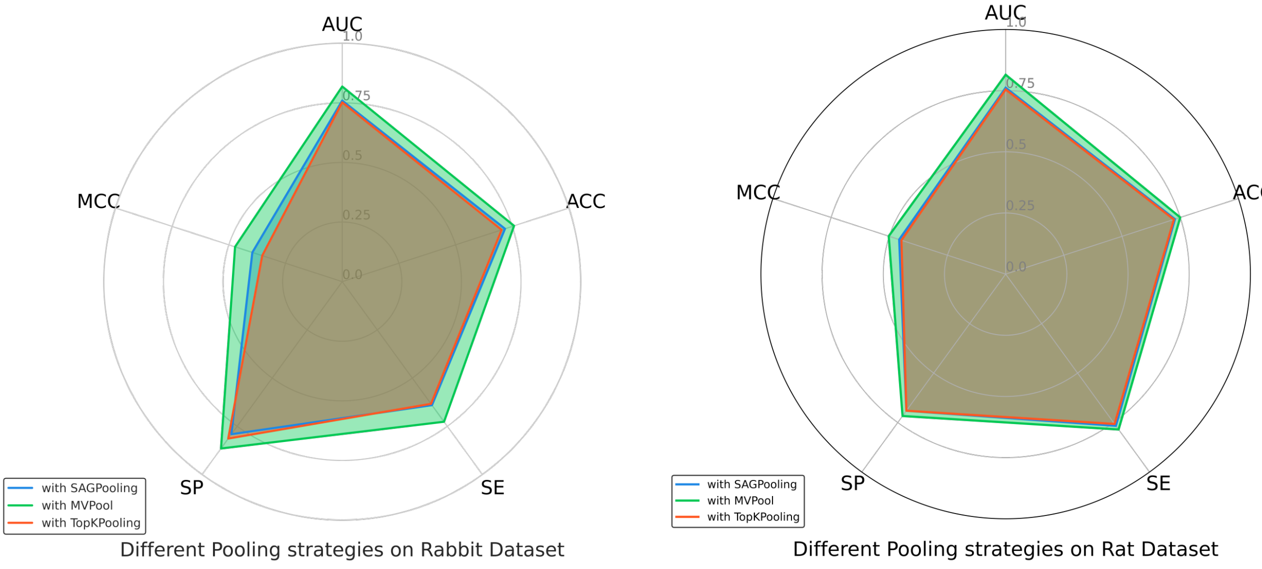

Figure S6: Model performance across various pooling strategy configurations.

14.3 Impact of Different Pooling Rates

Figure S7 displays model performance with varying pooling rates on the Rabbit and Rat datasets. Notably, between pooling rates of 0.2 and 0.8, increasing the rate significantly enhances model performance. Intuitively, higher pooling rates result in less retention of molecular graph structures. This indicates that compound ADT depends primarily on small, crucial substructures.

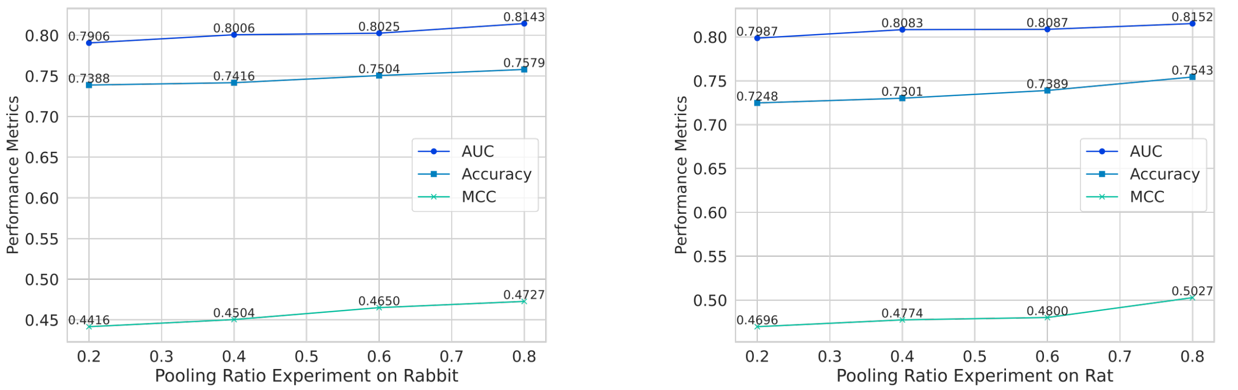

Figure S7: Model performance across various pooling rate configurations.

14.4 Impact of Different Learning Rates

Figure S8 illustrates the model's performance across various learning rates on the Rabbit and Rat datasets. The model performs stably at learning rates of 1e-2 and 1e-3, whereas at 1e-4 and 1e-5, performance deteriorates and becomes highly variable. This suggests that lower learning rates may impede model training.

14.5 Impact of Varying Hyperparameter λ

We investigated the impact of varying the hyperparameter λ on model performance. Specifically, with λ set between 0.1 and 0.9, we conducted multiple experiments to assess the model's performance. In these experiments, the Rabbit and Rat datasets were randomly split into training and test sets at a 9:1 ratio. Throughout each experiment, the training set, test set, and all other parameters remained consistent. The results are presented in Figure S9. It was observed that the model performance is stable when λ ranges from 0.1 to 0.9, with only a slight decrease between 0.3 and 0.9. Consequently, we can optimally set the hyperparameter λ to 0.3.

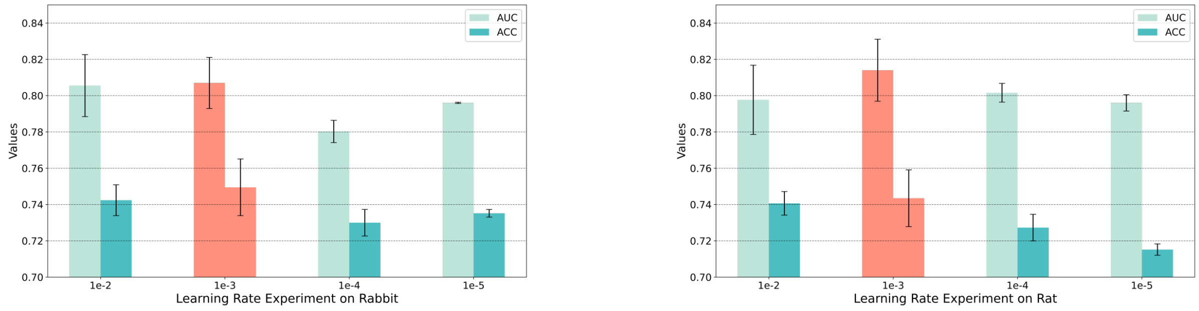

Figure S8: Model performance across various learning rate configurations.

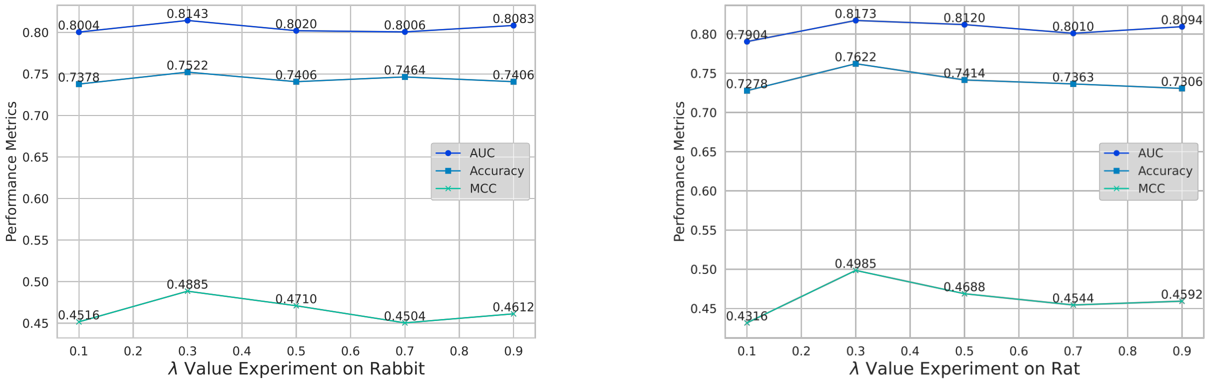

Figure S9: Model performance across various learning rate configurations.

15 Molecular Graph Representation Analysis

To investigate the graph representation capabilities of the proposed GraphADT model for learning compound molecules, we conducted a visual analysis using t-SNE[5] technology on both Rabbit and Rat test sets, as depicted in Figures S10(A), (B), (C) and (D). Compounds possessing ADT properties were labeled '1', while those lacking ADT properties were labeled '0'. As anticipated, compounds with similar ADT levels demonstrated greater similarity in feature space. Additionally, we compared the performance of the GraphADT and GIN models in learning the representation of compound molecular graphs. Figures S10(A) and (B) illustrate the prediction results of the GraphADT and GIN models on the Rabbit test set, respectively, while Figures S10(C) and (D) display the results for the Rat test set. The visualization results for the GraphADT and GIN models on both datasets significantly differed. Clearly, the GraphADT model was able to distinguish between ADT and non-ADT compounds effectively. Conversely, the GIN model struggled to accurately differentiate between ADT and non-ADT compounds. These findings demonstrate that the GraphADT model efficiently learns the molecular graph representations of compounds and accurately predicts their potential ADT properties.

16 Molecular Skeleton Alignment Analysis

To thoroughly analyze the performance of molecular graph representation learning, evaluating the quality of the representation space is essential. This study concentrates on a critical property influencing the quality of the representation space: alignment [Wang and Isola, 2020]. The fundamental concept of alignment is to map similar samples to comparable embedding vectors, essential for preserving the continuity and discernibility of molecular properties. To analyze the model's alignment in molecular representation, this study employed t-SNE technology to visualize compound molecules with varying skeletal structures. This aims to verify if the model can cluster compound molecules with similar structures into comparable representation spaces. In chemical research, the skeleton is the core structure of compound molecules and fundamental to studying their construction and interactions. Compound molecules with varying skeletons frequently exhibit substantial differences in chemical properties.

[5]https://en.wikipedia.org/wiki/T-distributed_stochastic_neighbor_embedding

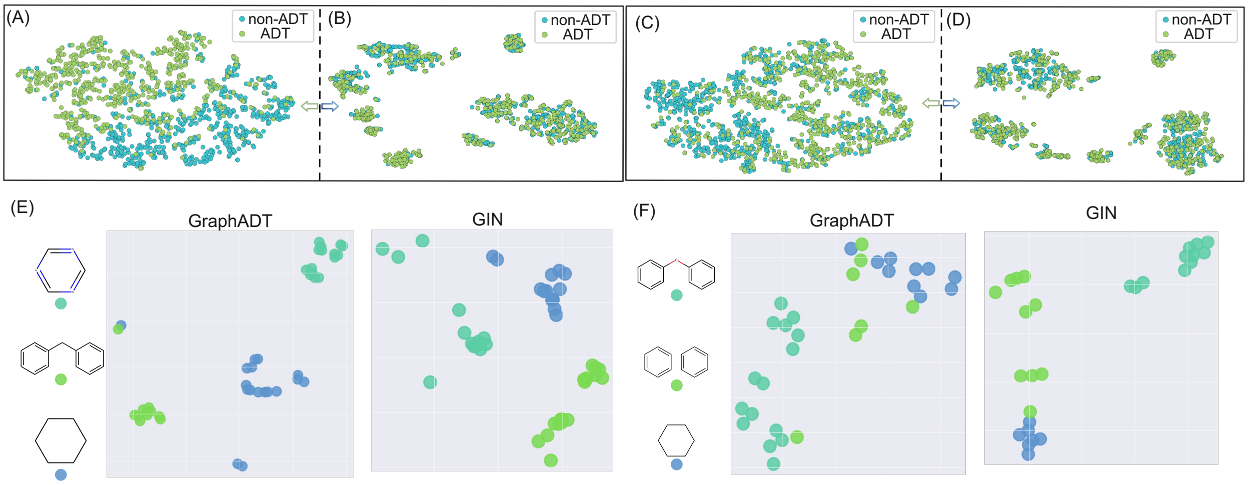

Figure S10: Molecular graph representation results of the (A) GraphADT and (B) GIN model on the Rabbit test set, and that of the (C) GraphADT and (D) GIN model on the Rat test set. Alignment results of the (E) GraphADT and (F) GIN model on the Rabbit test set, and that of the (C) GraphADT and (D) GIN model on the Rat test set.

Specifically, we selected three common skeletons from the Rabbit and Rat datasets, respectively, and marked them with distinct colors. As depicted in Figure S10(E) and (F), the GIN model fails to clearly distinguish between compound molecules with different skeletons. Conversely, the GraphADT model accurately projects compound molecules with identical skeletons into similar spaces, effectively distinguishing between molecules with different skeletons.

17 Discussion

Experimental results from public datasets demonstrate that the proposed GraphADT model outperforms current state-of-the-art models. The ablation study confirms the significant contributions of the structure remapped and multi-view graph pooling modules to the GraphADT model. Subsequently, t-SNE technology was employed to analyze the superior performance of the GraphADT model in molecular graph representation learning thoroughly. Additionally, through alignment analysis, it was demonstrated that the GraphADT model accurately projects compound molecules with identical skeletons into similar spaces, effectively distinguishing those with different skeletons. Finally, key atoms or substructures determining the compound's ADT were investigated using Shapley values, confirming the model's interpretability. Collectively, these results confirm that the GraphADT model effectively predicts the potential ADT of compounds and may guide the development of contact drugs and their treatments.

References

W. J. Adams, G. R. Biddinger, K. A. Robillard, and J. W. Gorsuch. A summary of the acute toxicity of 14 phthalate esters to representative aquatic organisms. *Environmental Toxicology and Chemistry: An International Journal*, 14(9):1569–1574, 1995.

B. Ballantyne. Acute percutaneous systemic toxicity of cyanides. *Journal of Toxicology: Cutaneous and Ocular Toxicology*, 13(3):249–262, 1994.

M. L. Berger and T. Sozeri. Rapid halogenated hydrocarbon toxicity in isolated hepatocytes is mediated by direct solvent effects. *Toxicology*, 45(3):319–330, 1987.

L. O. CHEMICALS. Globally harmonized system of classification and labelling of chemicals (ghs). 2001.

C. Cortes and V. Vapnik. Support-vector networks. *Machine learning*, 20:273–297, 1995.

Y. Dogra, A. G. Scarlett, D. Rowe, T. S. Galloway, and S. J. Rowland. Predicted and measured acute toxicity and developmental abnormalities in zebrafish embryos produced by exposure to individual aromatic acids. *Chemosphere*, 205:98–107, 2018.

J. G. Downing. Dermatitis from orthodichlorobenzene. *Journal of the American Medical Association*, 112(15): 1457–1457, 1939.

P. Ertl and B. Rohde. The molecule cloud-compact visualization of large collections of molecules. *Journal of Cheminformatics*, 4:1–8, 2012.

R. Feltens, I. Mögel, C. Röder-Stolinski, J.-C. Simon, G. Herberth, and I. Lehmann. Chlorobenzene induces oxidative stress in human lung epithelial cells in vitro. *Toxicology and applied pharmacology*, 242(1):100–108, 2010.

F. Harary and R. Z. Norman. Some properties of line digraphs. *Rendiconti del circolo matematico di palermo*, 9:161–168, 1960.

K. He, H. Fan, Y. Wu, S. Xie, and R. Girshick. Momentum contrast for unsupervised visual representation learning. In *Proceedings of the IEEE/CVF conference on computer vision and pattern recognition*, pages 9729–9738, 2020.

B. S. Hoppe, B. Laser, A. V. Kowalski, S. C. Fontenla, E. Pena-Greenberg, E. D. Yorke, D. M. Lovelock, M. A. Hunt, and K. E. Rosenzweig. Acute skin toxicity following stereotactic body radiation therapy for stage i non–small-cell lung cancer: who's at risk? *International Journal of Radiation Oncology* Biology* Physics*, 72(5):1283–1286, 2008.

G. Ke, Q. Meng, T. Finley, T. Wang, W. Chen, W. Ma, Q. Ye, and T.-Y. Liu. Lightgbm: A highly efficient gradient boosting decision tree. *Advances in neural information processing systems*, 30, 2017.

T. N. Kipf and M. Welling. Semi-supervised classification with graph convolutional networks. In *International Conference on Learning Representations*, 2016.

J. Lee, I. Lee, and J. Kang. Self-attention graph pooling. In *International conference on machine learning*, pages 3734–3743. PMLR, 2019.

L. Li, Z. Lu, G. Liu, Y. Tang, and W. Li. In silico prediction of human and rat liver microsomal stability via machine learning methods. *Chemical Research in Toxicology*, 35(9):1614–1624, 2022.

S. Lou, Z. Yu, Z. Huang, H. Wang, F. Pan, W. Li, G. Liu, and Y. Tang. In silico prediction of chemical acute dermal toxicity using explainable machine learning methods. *Chemical Research in Toxicology*, 2024.

A. Mathur, B. Gupta, A. Singh, M. Srivastava, and R. Raizada. Dermal toxicity of linear alkylbenzene sulfonate and quinalphos in guinea pigs. *Journal of Toxicology: Cutaneous and Ocular Toxicology*, 19(1):43–53, 2000.

A. Murray, E. Kisin, V. Castranova, C. Kommineni, M. Gunther, and A. Shvedova. Phenol-induced in vivo oxidative stress in skin: evidence for enhanced free radical generation, thiol oxidation, and antioxidant depletion. *Chemical Research in Toxicology*, 20(12):1769–1777, 2007.

S. J. Rigatti. Random forest. *Journal of Insurance Medicine*, 47(1):31–39, 2017.

T. Shen, Q.-X. Zhu, S. Yang, C.-H. Wu, H.-F. Zhang, C.-F. Zhou, and X.-J. Zhang. Trichloroethylene induced cutaneous irritation in balb/c hairless mice: histopathological changes and oxidative damage. *Toxicology*, 248 (2-3):113–120, 2008.

R. P. Sheridan, W. M. Wang, A. Liaw, J. Ma, and E. M. Gifford. Extreme gradient boosting as a method for quantitative structure–activity relationships. *Journal of chemical information and modeling*, 56(12):2353–2360, 2016.

P. Tomasulo. Chemidplus-super source for chemical and drug information. *Medical reference services quarterly*, 21(1):53–59, 2002.

P. Veličković, G. Cucurull, A. Casanova, A. Romero, P. Liò, and Y. Bengio. Graph attention networks. In *International Conference on Learning Representations*, 2018.

T. Wang and P. Isola. Understanding contrastive representation learning through alignment and uniformity on the hypersphere. In *International conference on machine learning*, pages 9929–9939. PMLR, 2020.

K. Xu, W. Hu, J. Leskovec, and S. Jegelka. How powerful are graph neural networks? In *International Conference on Learning Representations*, 2018.

Z. Ying, J. You, C. Morris, X. Ren, W. Hamilton, and J. Leskovec. Hierarchical graph representation learning with differentiable pooling. *Advances in neural information processing systems*, 31, 2018.
